# Case of Severe Compound Fracture of Both Bones of the Leg

**Published:** 1842-05-14

**Authors:** E. Hartshorne

**Affiliations:** Resident Surgeon


### CLINICAL REPORTS.

### *Pennsylvania Hospital—Surgical Wards—Service of Dr. Norris.*

By E. Hartshorne, M. D., Resident Surgeon.*

*Case of Severe Compound Fracture of both bones of the Leg.*—Wm. M., æt. 32, of sanguine nervous temperament, not very muscular, but in good health, and temperate, was admitted July 31st, with a bad compound and double fracture of both bones of the right leg, received the day previous at Havre de Grace.

This was a case of very serious injury, resulting from the caving in of a heavy bank of clay, ten feet in height, upon the man while he was digging at its base. He was violently thrown on his right side, with the left foot under the right leg. This latter sustained the whole force of the blow and the pressure of the superincumbent weight, while the former, together with the left leg, escaped unhurt. The entire body also, except the head and shoulders, was buried under the fallen mass, but did not undergo any severe contusion.

The fractures were reduced, the wound covered with bran,—its edges having been brought together with sutures and adhesive strips,—and the limb well fixed in a fracture box with cotton and bran, about an hour after the accident, by a practitioner of the neighbourhood. This dressing was so securely and comfortably applied, that when the patient was transported the next morning on the rail road, sixty miles to Philadelphia, he suffered comparatively little pain; nothing but the pressure on his heel giving him material uneasiness. He entered the hospital twenty-four hours after the reception of the injury.

The right tibia and fibula were then ascertained to be rather obliquely fractured about half way between their epiphyses, and again about three and

* This, and the case last reported, were treated under the direction of Dr. E. Peace. In narrating the last case, p. 234, the reporter omitted to state that the exposed bone was excised from the upper fragment.

a half inches from the ankle. Near the seat of the lower breach of continuity the fragments had been forced through the integuments on the inner side, so as to produce an irregularly semicircular lacerated wound, extending from within three inches of the internal malleolus obliquely upwards at least five inches. The rest of the leg and foot, and especially the external malleolus, had been much contused, and were then in a state of violent inflammation. Although the bones were presumed to be splintered, no small fragments could be found entirely loose, and the patient was not aware that any spicula had been removed at the first dressing.

The limb was extended in a fracture box on a pillow covered with oil cloth, as is usual in such cases; iced water was constantly applied to the inflamed surface; and the wound, which looked irritated and dirty, was relieved of its sutures and adhesive strips, cleansed, and covered with a warm poultice. An opiate was then administered, and directed to be repeated every night, or oftener if required. During the succeeding four days it was necessary to use the catheter, the man being unable, while on his back, to empty his bladder. The cold application was discontinued in a few days, as soon as the most active inflammation had subsided. Pledgets of lint and charpie were subsequently laid along the limb at each dressing, in order to absorb and retain the discharge. They were changed twice a day with the poultices until bran was employed.

Suppuration appeared to be fully established on the third day, and soon became very abundant; the pus flowing copiously from abscesses opening into the wound on every side, and extending in all directions around it. The discharge before long assumed an unhealthy character, becoming dark, thin, flakey and fetid; and it continued more or less so four or five weeks. This secretion was particularly free from the lower margin of the wound, where it was frequently accompanied with the flow of a serous fluid, like synovia, issuing from a cavity apparently in communication with the joint, extending in front of the ankle and instep and on their inner side, in which region even gentle pressure caused acute pain. A superficial slough of moderate extent appeared on the external malleolus; this was poulticed for two or three weeks, and then dressed as a simple ulcer, under which treatment the denuded surface readily cicatrised. Notwithstanding the free evacuation of the pus through numerous openings in the original wound—assisted by the application of warm poultices twice daily, and as much expression, at least every morning, as the patient in his reduced and irritable state could safely bear,—yet extensive burrowing took place among the muscles of the calf and the extensors. New abscesses successively occurred; the matter approached the surface at different points in front and outside near the upper seat of fracture, and was there allowed an exit by repeated counter-openings. This exhausting drain, the protracted suffering, and their accompanying constitutional irritation, prostrated the patient to an alarming degree, although he was faithfully supplied with the most nutritious diet and frequent draughts of porter. Such, indeed, continued to be his condition, generally and locally, that three, if not four weeks elapsed, before it entirely ceased to be a matter of deliberation from day to day whether or not secondary amputation would have to be performed. By that time the discharge had become perceptibly more healthy and less profuse; the patient suffered less pain at the dressing, and granulation advanced more rapidly and firmly in the wound, while most of the sinuses began to close. The poultice to the ulcer was replaced by less relaxing applications, and these again, by degrees, were made more stimulating and astringent.

Increased attention was then paid to the accurate adjustment of the fragments, and their retention at rest in position. The latter was effected by means of compresses carefully disposed on each side of the limb within the pillow, the sides of the fracture box being brought nearer together, so as to exert greater pressure. These indications were, of course, by no means disregarded even at the outset; but they were necessarily much more easily and fully answered at a later period in the treatment of the case.

The patient's sufferings were seriously aggravated by the unusual persistence and severity of burning pain in the heel from pressure, despite of varied and unceasing efforts to obviate the latter. None of the ordinary expedients, such as suspending the foot, supporting the ankle, or the extreme point on the sides of the heel, afforded any permanent relief; nor did any plan devised serve to prevent the occurrence of a small ulcer on the part.*

After the pillow and compresses had been employed two months, during which time the pillow was renewed, on account of its soiled condition, at least twice, they were replaced by the bran dressing, arranged in the following manner. The floor and open angles of the box having been covered with a layer of cotton, upon this was placed a piece of oiled linen large enough to fit in and line the whole interior of the instrument. A quantity of bran sufficient to make a comfortable, firm, and uniform bed for the limb, was next thrown in. On this bed the limb was then placed, without any additional support under the heel or elsewhere, and the apparatus closed up; more bran being packed in along the sides and filling up all the irregularities of surface, so as to press equably on every part. The benefits of this change manifested themselves so promptly and decidedly, as to excite regret that it had not been an earlier resort. The man expressed entire relief from pain, and especially in the heel, which now remained permanently at ease. The ulcer on its posterior face, which had yielded very little to a variety of applications,—pressure being always as much as possible avoided,—soon began to cicatrise, and gave no further trouble. The flow of pus lessened, the wound fast disappeared, consolidation rapidly proceeded, and the man's health daily improved.

Perfect rest was secured to the limb for three weeks; the bran, which became moistened by the still prevalent discharge, being replaced from time to time with a fresh article. At the end of this period union was found to be so far advanced, that the patient was able, unaided, to elevate his leg. The box was discontinued at the commencement of the twelfth week, a roller having been firmly applied in its stead. The wound entirely healed by the fourteenth week. Small fistulous openings continued to afford issue to matter on the outside of the leg near the upper seat of fracture. Paste board splints were put on at that time, and the patient was allowed to leave his bed. His health was then completely restored, and he had become quite fleshy and florid. He had not been walking about longer than two weeks before he was seized with violent erysipelas of the leg. This new affliction was preceded and accompanied by severe constitutional symptoms, of which

* Unfortunately, neither the air nor the water cushion, as commonly prepared, were within reach on this occasion; a good bladder, however, might have been resorted to for either purpose.

the cerebral, as evinced by intense cephalalgia, delirium, and stupor in succession, were strongly marked.

The general treatment for this new complication consisted in antiphlogistic measures at the onset, subsequently mild stimulations, with wine whey, beef essence, and cold infusion of cinchona; calomel in alterative doses, urged to ptyalism, and revulsion by a blister to the back of the neck. Cold slippery elm mucilage was applied to the affected part. Velpeau's solution of the sulphate of iron was tried and abandoned as inefficient in this case. Tincture of iodine and nitrate of silver were used in succession, but without avail, to circumscribe the eruption. This latter by degrees enveloped the whole limb, involving the lymphatic ducts and glands. It subsided with the constitutional symptoms in about two weeks, but was succeeded, however, by a deep seated abscess in the thigh, which discharged pus largely for about a week, and then closed. Meanwhile, during the progress of the second convalescence, the fistulous sinuses in the leg became obliterated without the exfoliation of any bone. Discharged Feb. 23d, cured,—some rigidity of the ankle joint still persisting; the injured leg, however, being very nearly if not quite as straight as the sound one, and not deformed by any tumours of callus.

*Remarks.*—The foregoing history affords an interesting proof of the entire adequacy of the simplest means to complete the cure of one of the worst forms of accidental injury which the practitioner is ever called upon to treat. The successful termination of a case so doubtful in its issue, and so far involving the safety of life and limb, amply repays the surgeon for any amount of pains bestowed upon the treatment, while it tends much to increase his confidence in the recuperative powers of nature when well seconded by the various appliances of therapeutic science and art.

The age, health, and good habits of this patient suggested a favourable prognosis, and induced the attempt to save a mangled extremity that must have been at once removed, had it been crushed at sea or on the battle-field; and which under the happiest auspices presented a condition sufficiently discouraging to make the boldest and most experienced surgeons pause ere they resolved to risk a life for the salvation of a member. The man seemed to be peculiarly alive to pain, which he was ill qualified to endure; and the intense suffering, almost inseparable from so grave an injury, soon appeared to deprive him of the little fortitude he at first possessed. Nevertheless, after having passed the dangers of gangrene, always to be dreaded for some days after such an accident, he escaped those of constitutional irritation; of absolute prostration from the exhausting suppuration, with its frequently attendant hectic fever, colliquative sweats and diarrhœa; of erysipelas; of purulent absorption; and lastly, of pneumonia. He was spared from all but the least fatal of these enumerated complications, erysipelas; nor did this manifest itself until the man had been some time convalescent. It is worthy of remark, by the way, that although the erysipelas was unusually violent in character, and was followed by a large phlegmonous abscess in the thigh, yet no softening from absorption of the callus, which not unfrequently results from such a cause, occurred in the seat of either fracture. The two fistulous sinuses still open near the upper fracture, gave exit to an increased flow of pus during the persistence of the erythema, but closed entirely soon after the subsidence of the latter, and cicatrised without the loss of a particle of bone. The employment of bran appears to have answered a decidedly good purpose in the management of this case. It fully proved its superiority when compared with the apparatus first adopted. No comparison was instituted between it and the treatment by suspension of the whole limb, which is sometimes resorted to when the heel is troublesome, and especially when the wound is not very accessible from above. Such a trial was rendered unnecessary by the entire success of the more convenient plan.

